# Effects of depression on patients suffering from ankylosing spondylitis: a comparative study

**DOI:** 10.1590/1516-3180.2024.0177.R1.16102024

**Published:** 2025-04-04

**Authors:** Antonio Cortés-Rodríguez, Lisa Alves-Gomes, Marta Losa-Iglesias, Juan Gómez-Salgado, Ricardo Becerro-de-Bengoa-Vallejo, Miguel Ángel Saavedra-García, Daniel López-López, Ana María Jiménez-Cebrián

**Affiliations:** I Department of Health Sciences, Faculty of Nursing and Podiatry, Universidade da Coruña, Industrial Campus of Ferrol, Ferrol, Spain.; II Full Professor, Nursing School, Minho University, Braga, Portugal. Nursing Research Center (CIEnf) of the University of Minho; Health Sciences Research Unit: Nursing (UICISA: E), Nursing School of Coimbra, Portugal.; III Full Professor, Department of Nursing and Stomatology, School of Health Sciences, Universidad Rey Juan Carlos, Alcorcón, Spain.; IV Department of Sociology, Social Work and Public Health, Faculty of Labour Sciences, University of Huelva, Huelva, Spain; Safety and Health Postgraduate Programme, Universidad Espíritu Santo, Guayaquil, Ecuador.; V Full Professor, Department of Physiotherapy and Podiatry, School of Nursing, Universidad Complutense de Madrid, Madrid, Spain.; VI Physical Education and Sports Department, Group of Research in Sport Science, Universidade da Coruña, A Coruña, Spain.; VII Research, Health and Podiatry Group, Department of Health Sciences, Faculty of Nursing and Podiatry, Universidade da Coruña, Industrial Campus of Ferrol, Ferrol, Spain.; VIII Department of Nursing and Podiatry, Faculty of Health Sciences, University of Málaga, Málaga, Spain.

**Keywords:** Comorbidities, Anxiety, Behavioral symptoms, Bone diseases, Beck depression inventory, Psychological assessment, Chronic inflammatory diseases

## Abstract

**BACKGROUND:**

Ankylosing spondylitis (AS) is a sustained inflammatory pathology that manifests as increasing rigidity and a continuous decline in spinal flexibility, leading to increasing lumbar pain during rest.

**OBJECTIVES:**

This study primarily aimed to evaluate depression assessments using the Beck Depression Inventory (BDI) and delineate depressive symptomatology in patients diagnosed with AS compared to those without this condition.

**DESIGN AND SETTING:**

A comparative study was conducted in Medical Centers in Málaga, Spain.

**METHODS:**

A cohort of 102 participants, with a mean age of 46,80 ± 10,54 years, was divided into two sets: 51 individuals diagnosed with AS (cases) and another 51 without AS (controls), each harmonized across variables such as body mass index, age, and sex. Demographic variables were systematically gathered from each participant, and the BDI responses were accurately recorded and subsequently analyzed for comparison.

**RESULTS:**

Of the total sample, the sex distribution was 29.4% male and 70.6% female. BDI scores were higher for the AS group (19.25 ± 15.5) than for the control group (5.33 ± 7). Notably, there were clear statistical differences (P < 0.01) in the BDI categories, with elevated levels observed in participants with AS.

**CONCLUSION:**

Individuals with AS experienced higher levels of depression than those without AS. Furthermore, there were sex differences within the case group, with a higher percentage of women than men at any level of depression. Notably, there was a moderate inverse correlation between the number of years since diagnosis and depression level.

## INTRODUCTION

Ankylosing spondylitis (AS) is a persistent inflammatory disorder predominantly affecting the axial skeleton, marked by symptoms such as inflammatory back pain, rigidity, and gradual reduction in spinal flexibility.^1,2^ The prevalence of AS in Spain is estimated to be 0.26%, similar to that found in other European Union countries, although, globally, there is considerable variation between countries.^3^

AS more commonly affects men than women, with a ratio of approximately 2-3:1 in radiographic AS.^4^ The epidemiology of AS varies by geographic region, with a prevalence ranging from 0.1% to 1.4% in Europe, and is higher in populations with a high prevalence of HLA-B27, such as in Northern Europe and certain ethnic groups.^1^ In addition, it has been observed that women with AS receive more diagnostic codes than men, including a higher coding of peripheral symptoms and a higher prevalence of diagnostic codes for depression.^5^

Depression in AS has been studied in various ways, as a symptom of the disease^6,7^ or as a cause of it.^8^ Considering that AS affects the axial skeleton, causing pain in the spine and leading to lumbar pain, mobility restriction, and even sleep problems,^9,10^ depression is acknowledged as a significant comorbid factor in patients with AS, exerting a detrimental influence on both the quality of life and clinical trajectory of the disease.^11^

Subjects with AS demonstrate a significantly higher prevalence of depression compared to the healthy population, which substantially degrades their quality of life and aggravates the severity of their clinical outcomes.^12^ Moreover, significant sleep disruptions are frequently identified in patients with AS, potentially correlated with pain and additional symptomatic expressions of the disease.^13^ The incidence of depression diagnosed in those with AS is approximately 80% greater in females and 50% greater in males than in the general population.^14^ Another study in Korea revealed that post-diagnosis depression risk among individuals with AS was 2.21 times higher than in a control group, with a higher risk in female patients, older patients, those with low socioeconomic status, and those with chronic comorbidities.^15^

Currently, there is a lack of research in Spain that analyzes depression severity considering its multifaceted nature, including affective, behavioral, and cognitive dimensions, as well as anxiety, in patients with AS.

Fatigue, a common multidimensional symptom in AS, is significantly associated with disease activity and depression, highlighting the need to address psychogenic elements, particularly depression, in the treatment of AS.^16^ Additionally, considerable sleep alteration has been reported in these patients, which could be strongly linked to pain and other symptoms of the disease.^13^ Consequently, the review of the presented literature has identified the lack of existing data on the comprehensive assessment of depression between AS-diagnosed and non-diagnosed respondents in comparative studies in the Spanish context. Therefore, this study aims to contribute to the improvement of AS-affected individuals’ health and quality of life by comparing depression risk between patients with AS and healthy controls and establishing a case-control study design.

It is hypothesized that patients diagnosed with AS will exhibit significantly higher levels of depression, as measured by the Beck Depression Inventory (BDI), than a control group without AS and that this depression will have a detrimental impact on their overall quality of life.

## OBJECTIVES

The principal aim of this study was to evaluate depression assessments using the BDI to delineate depressive symptomatology in patients diagnosed with ankylosing spondylitis (AS) compared to those without this condition.

## METHODS

### Sample Design

A sample of 102 individuals was selected using a voluntary, systematic, and non-random sampling approach. This methodology was selected because of the voluntary nature of participant recruitment. This approach ensured the inclusion of patients with AS.

The study cohort was divided into two groups: 51 patients with AS diagnosed via X-ray confirmation by a rheumatologist and 51 control participants without AS. Recruitment was conducted between December 2022 and June 2023. Patients with AS were recruited through patient organizations in Sevilla and Córdoba, Spain, while control participants were sourced from the podiatry departments of the Policlínica Lacibis and Alhaurín Torre Salud medical centers.

The inclusion criteria required participants to be legal adults, capable of providing informed consent, and clearly distinguished as AS or non-AS. Control participants were matched to the AS group based on age and sex. Those who failed to meet these criteria and were unable to independently undertake activities of daily living or something similar were excluded.^17^ Specifically, three control participants were excluded for not meeting the matching criteria, one individual was excluded for failing to indicate their sex, and two were excluded due to significant age differences compared to the AS group. This study adhered to the Strengthening the Reporting of Observational Studies in Epidemiology (STROBE).^18^

### Sample Size Calculation

To determine the appropriate sample size for this case-control study, Epidat software 4.2 was employed. This tool was developed by the Consellería de Sanidade of Xunta de Galicia, Spain, in collaboration with the Pan-American Health Organization (PAHO-WHO) and Universidad CES of Colombia. The sample size was estimated based on an assumed confidence level of 75%, statistical power of 0.80, odds ratio of 2.0, and anticipated exposure proportions of 50% in the cases and 33.333% in the controls. Consequently, the study had a sample size of 102 participants, with an equal distribution of 51 individuals in each group.

### Procedure

In research on the relationship between AS and depression, data collection included sociodemographic variables such as sex, age, height, weight, body mass index (BMI), comorbidities, employment status, educational level, and marital status.

The study participants, diagnosed with AS, completed the BDI, a validated questionnaire^19,20^ and translated into Spanish; the BDI is recognized for its effectiveness in assessing depression symptoms.^21,22^ This instrument consists of 21 items, each with a score of 0 to 3 points, with a total possible score of 63 points. Outcomes are classified into multiple ranges: absence of depression (0-9 points), mild depression (10-15 points), moderate (16-23 points), and severe (24-63 points).

The BDI is notable for its high reliability, demonstrated by a Cronbach’s alpha coefficient of 0.85 to 0.889, and is suitable for both psychiatric and non-psychiatric patients, distinguishing between subtypes of depression and depression from anxiety.^21^ This interculturally applied questionnaire evaluates a wide range of symptoms, including mood disorders, loss of hope, feelings of guilt, and fatigue, among others. The BDI score is a valuable indicator of the need for professional intervention, especially for scores above 17.^19,20,21,22,23,24^

This study offers a comprehensive view of the mental state of patients with AS and contributes to our understanding of the interactions between this chronic disease and mental health.

### Ethical Considerations

This study was approved by the Ethics Committee for Experimental Research at the University of Málaga, Spain, and was assigned identification number 122-2022-H on February 2, 2023. All the methods and procedures were conducted in strict accordance with the ethical guidelines of the Declaration of Helsinki.^25^

### Statistical Analysis

Sociodemographic data were analyzed, including variables such as sex, age, height, weight, and BMI, along with other independent variables. These are expressed as the mean and standard deviation (SD), and the maximum and minimum values are provided. To assess the normality of the data, we employed the Kolmogorov-Smirnov test, considering data distributions as normal if P-values exceeded 0.05. The results indicated non-normal distributions for the study variables, as demonstrated by P values less than 0.05, prompting us to apply the Mann-Whitney U test to detect statistically significant differences between groups.

Frequencies and percentages were calculated for categorical data. We used the chi-square test to compare the differences between the two groups in the BDI category. The relationship between the number of years since diagnosis and the degree of depression was evaluated using Spearman’s correlation.

All statistical evaluations were performed using SPSS software version 27.0.1.0 (IBM-Corporation, Armonk, NY, United States).

## RESULTS

### Description and Comparison Data

The data revealed a non-normal distribution (P < 0.05) for all analyzed variables (age, weight, height, BMI, and BDI scores). The study was conducted with a sample of 102 subjects divided into two groups of 51 each: a case group (AS) and a control group. Participants from both cohorts were matched based on sex, age, and BMI. As shown in **Table 1**, no statistically significant differences were observed (P > 0.05), except at the time of diagnosis because the control group did not have AS. All participants exhibited the characteristics outlined in **Table 1**. The mean duration since the diagnosis of AS was considerably high (11.46 years), although the data demonstrated notable variation as shown by the SD (± 11.51 years) with a range of 0.3 to 50 years.

**Table 1:** Descriptive and comparative data (Spain, 2023)

Descriptive data		Total (n = 102)	AS (n = 51)	Control (n = 51)	P value*
		Mean (SD)	Mean (SD)	Mean (SD)	
Age (years)		46.8 0 ± 10.54 (24-70)	46.45 ± 11.38 (24-70)	45.88 ± 9.73 (28-68)	0.683^†^
Weight (kg)		70.70 ± 15.09 (44-115)	71.61 ± 17.01(44-115)	69.79 ± 13 (52-102)	0.886^†^
Height (m)		1.67 ± 0.08 (1.5-1.88)	1.67 ± 0.08 (1.5-1.86)	1.67 ± 0.09 (1.52-1.88)	0.644^†^
BMI (kg/m^2^)		25.37 ± 5.05 (17.04-44.44)	25.67 ± 5.74 (17.04-44.44)	25.08 ± 4.28 (19.33-39.84)	0.776^†^
Sex (%)	Male (%)	30 (29.4%)	15 (29.4%)	15 (29.4%)	1^‡^
	Female (%)	72 (70.6%)	36 (70.6%)	36 (70.6%)	
Time since AS diagnosis (years)		N/A	11.43 ± 11.51 (0.3-50)	N/A	< 0.01^†^

BMI = body mass index; SD = standard deviation; AS = ankylosing spondylitis.

*In all analyses, P < 0.05 (95% confidence interval) was considered statistically significant.

†The Mann-Whitney U test was applied.

‡ Frequencies (percentages) and the chi-square (X^2^) test were employed.

Comorbidities were assessed among the participants. Various comorbidities were identified, including hypertension, diabetes, multiple sclerosis, heart disease, fibromyalgia, and vascular insufficiency, each represented by an individual case within the sample. This corresponds to 1% of the participants in each condition. Because of the diversity and low frequency of these specific comorbidities, they were not incorporated in detail in the main analysis.

Furthermore, a higher proportion of the total sample (16.66%) was obese. Specifically, in the case group, a higher prevalence of obesity was noted (15.68%) than in the control group (11.76%). The data on obesity in patients with AS were similar to those of other studies that have specifically investigated obesity.^26^

**Table 2** shows the results of the educational level analysis. A varied distribution of educational levels was observed in both the entire sample and in the specific case and control groups. The most represented category in the total sample was the third level of study, accounting for 36.27% of the participants, followed by individuals with higher education (24.51%). When analyzing the groups separately, 39.22% of participants in the AS group and 33.33% in the healthy group were categorized as third-level studies. Furthermore, in both the case and control groups, individuals with higher education represented approximately a quarter of each group, with 23.53% and 25.49%, respectively. The primary and secondary levels were similar in both groups. It is noteworthy that the chi-square test demonstrated a lack of statistically significant discrepancies in the distribution patterns of educational attainment levels when comparing the case and control cohorts.

**Table 2:** Descriptive and comparative of education levels (Spain, 2023)

Education Level	Total (n = 102)	AS (n = 51)	Control (n = 51)	P value*
Incomplete Primary Level	37 (36.3%)	20 (39.2%)	17 (33.3)	‡0.983407
Primary Level	19 (18.6%)	9 (17.6%)	10 (19.6%)	‡0.983407
Secondary Level (High School)	19 (18.6%)	9 (17.6%)	10 (19.6%)	‡0.983407
Third level (3-years university studies)	37 (36.3%)	20 (39.2%)	17 (33.3)	‡0.983407
Higher Studies (university studies of 5 years or more)	25 (24.5%)	12 (23.5%)	13 (25.5%)	‡0.983407

‡ Chi-square (X^2^) test was utilized. AS = ankylosing spondylitis.

### Outcome Measurements

**Table 3** presents the statistically significant differences in the BDI scores between the two groups. Subjects with AS exhibited higher scores (BDI = 19.25 ± 15.50), in contrast to the lower scores of the control group (BDI = 5.33 ± 7). Statistically significant differences were observed in the BDI categories between the AS and control groups, as indicated in the same table. Notably, severe depression was exclusively observed in the AS cohort.

**Table 3:** Relationship between Beck Depression Inventory scores and categories among patients with ankylosing spondylitis and the control group

Outcome Measurements		Total group Mean ± SD (range) n = 102	AS group Mean ± SD (range) n = 51	Control group Mean ± SD (range) n = 51	P value (Cases vs. Controls)
BDI category*	No Depression	51 (50%)	9 (17.65%)	42 (82.35%)	**0.002‡** **0.67**
	Mild	18 (17.65%)	13 (25.49%)	5 (9.8%)	
	Moderate	16 (15.69%)	12 (23.53)	4 (7.84%)	
	Severe	17 (16.67%)	17 (33.33%)	0 (0%)	
BDI scores		12.29 ± 14.75 (0-40)	19.25 ± 15.50 (0-40)	5.33 ± 7 (0-18)	**< 0.001†**

*BDI = Beck Depression Inventory; SD = standard deviation; AS = ankylosing spondylitis. Frequency, percentage (%), and the chi-squared test (‡) were employed. The BDI domains are categorized as follows:

(1) 0-9 points indicate no depression, (2) 10-15 points indicate mild depression, (3) 16-23 points indicate moderate depression, and (4) 24-63 points indicate severe depression. † BDI scores, median, interquartile range, range (min–max) and Mann–Whitney U test applied. For all analyses, P < 0.05 (within a 95% confidence interval) was considered statistically significant (in bold).

**Table 4** displays the correlation between the duration since AS diagnosis and BDI scores. Since the BDI Score data did not show a normal distribution and the relationship between the two variables may not be linear, the correlation was assessed using the Spearman coefficient (-0.331). This moderate negative correlation indicated an inverse relationship between the two variables.

**Table 4:** Correlation between time since diagnosis and degree of depression

	Time since AS diagnosis (years)	P value
BDI* Scores	-0.331†	**0.018**

*BDI = Beck Depression Inventory; AS = ankylosing spondylitis.

†Spearman’s rho. In all analyses, P < 0.05 (within a 95% confidence interval) was regarded as statistically significant (in bold).

## DISCUSSION

The main objective of this study was to compare BDI scores to categorize depression severity in subjects diagnosed with AS and an unaffected group in Spain.

This case-control study was conducted to examine the impact of depression in individuals with AS and healthy individuals. After reviewing the scientific literature, we found insufficient studies measuring the influence of anxiety and depression on these patients in Spain.

The results indicate that the majority of subjects with AS in this study experienced depression (82.35%) at one of its three levels, compared to the control group (17.65%), data that are in line with levels similar to other studies conducted with the BDI,^27,28^ and higher than studies that used other tools to assess depression.^29,30^

A recent study aimed to evaluate depression in patients with AS with a gender focus, determining that, in relation to depression, there were factors that affected depression differently; for example, pain was a greater determinant in women than in men.^31^ In our study, approximately 46.67% of men and 97.22% of women with AS showed some degree of depression, values very similar to those obtained by Meesters et al., although it was concluded that the rate of depression was higher in women in the general population.^14^

Depression has also been analyzed in patients with AS, considering their level of education or occupational status, with varying results. In the study conducted by Karetekin et al., these two parameters were not considered statistically significant.^7^ However, Kilic et al. considered the educational level to be a determining factor associated with depression, noting that lower levels had a higher probability of depression.^32^ In the analysis of the relationship between the level of education and the prevalence of different degrees of depression in the case group of our study, no clear trend is identified, suggesting that a higher educational level leads to a lower probability of suffering from depression. For instance, participants with incomplete primary education showed a 100% prevalence of mild depression, whereas those with higher education exhibited a 41.67% prevalence of severe depression, which was considerably higher than those with other educational levels. Moreover, in the tertiary education category, the percentage of depression was more evenly distributed across different degrees and did not show a significant decrease in severe depression with increasing educational level. These findings suggest that the relationship between educational level and depression might be influenced by multiple factors and that education alone is not a clear predictor of the risk of depression in individuals with AS.

Because AS is a chronic disease,^1^ the evolution of depression over the years was analyzed in this study, obtaining results that suggest that, on average, the longer the duration of the disease, the lower the level of depression (**Table 3**). These results are similar to those obtained in a previous study that evaluated depression levels over 15 years and found that depression decreased over time in patients with AS.^7^

In our study, the range of depression was assessed using the BDI questionnaire. This document was validated in Spanish by other authors,^21,22^ and utilized to assess depression in patients with AS.^12,33,34^ Previous research on depression in chronic diseases has supported the use of the BDI as an effective self-assessment tool for the detection and monitoring of depression in patients with multiple sclerosis^35^ or Parkinson’s disease,^36^ the latter demonstrating that patients with Parkinson’s disease matched healthy subjects and found that depression constitutes a significant risk for increased symptoms and adverse effects on their health. It has also been used in musculoskeletal pathologies such as subacute back pain,^37^ which is a frequent symptom in patients with AS.^1,2^ Our study is in line with previous research that examined special population groups related to depressive symptoms in chronic illnesses.^38,39^ Populations such as those with rheumatic diseases and chronic pain syndromes exhibit a high prevalence of depressive symptoms,^39^ emphasizing the need to assess and address depression in chronic inflammatory conditions such as AS.^11^

This study had several limitations. First, it was not a randomized controlled trial. Future studies could have a more diverse sample size, including subjects from other regions or countries, to improve the robustness of the study, as differences have been evidenced according to origin.^30^ Additionally, in relation to depression, it would have been interesting to consider more factors to assess depression, such as the disease activity index (BASDAI) or sports practice; the latter has shown a decrease in depression levels in patients with AS.^34,40^

Considering the findings of this study, it would be valuable to develop new avenues of interventional research to address depression as an additional AS symptom. Among these, Mindfulness-Based Stress Reduction therapies are promising alternatives. These therapies have already shown significant improvements in anxiety and depression levels in individuals with chronic diseases, particularly during the COVID-19 pandemic.^41^ Integrating such interventions into the treatment of AS could positively impact patients’ quality of life and should be explored in future studies.

## CONCLUSIONS

Higher scores and ranges of depression were observed in subjects with AS matched with healthy subjects; therefore, we can affirm that they have a higher risk of depression and should be monitored. The degree of depression in subjects with AS decreases with time.
